# Efficacy of caudal epidural injection of Xylazine-Lidocaine HCl and detomidine-lidocaine HCl in domestic horses (*Equus ferus caballus*)

**DOI:** 10.1186/s12917-025-04840-7

**Published:** 2025-05-28

**Authors:** Adel Sobhy, Ahmed G. Nomir, Mohamed A. Hamed, Mohamed M. A. Abumandour, Mahmoud El-Kammar

**Affiliations:** 1 Department of Surgery, Faculty of Veterinary Medicine, Damanhour University, Damanhour, 22516 Egypt; 2 Department of Anatomy and Embryology, Faculty of Veterinary Medicine, Damanhour University, Damanhour, 22516 Egypt; 3 Department of Surgery, Anesthesiology and Radiology, Faculty of Veterinary Medicine, Aswan University, Aswan, Egypt; 4 Department of Anatomy and Embryology, Faculty of Veterinary Medicine, Alexandria University, Abees 10th, P.O. 21944, Alexandria, 21944 Egypt; 5 Department of Surgery, Faculty of Veterinary Medicine, Alexandria University, Abis 10, P.O. 21944, Alexandria, Egypt

**Keywords:** Equine, Xylazine-Lidocaine HCl, Detomidine-Lidocaine HCl, Perineal analgesia and sedation, Epidural anesthesia

## Abstract

The current study was prepared to compare the efficacy of xylazine-lidocaine HCl and detomidine-lidocaine HCl following caudal epidural injection in horses, evaluating sedation, analgesia, physiological parameters, and hemato-biochemical values. This study was applied to six healthy adult horses (300–350 kg, > 4 years of age). The horses were randomly divided into two equal groups. Group 1 (seven horses) received xylazine (0.17 mg/kg bwt) + lidocaine HCl (0.06 mg/kg bwt), while Group 2 received detomidine (0.03 mg/kg bwt) + lidocaine HCl (0.06 mg/kg bwt) via caudal epidural injection. Perineal analgesia and sedation (onset time and duration) were assessed before administration and at 15, 30, 60, 90, and 120 min post-administration. Concurrently, heart rate, respiratory rate, rectal temperature, and hemato-biochemical values were recorded. The sedative duration of detomidine was longer than that of xylazine (94 ± 0.96 vs. 85 ± 0.94 min). Both detomidine and xylazine induced complete bilateral perineal analgesia in all horses. Analgesia onset was slightly faster and duration longer in detomidine-treated horses compared to xylazine, and these values are for onset of analgesia (11.79 ± 1.15 vs. 14.46 ± 0.92 min). Significant heart rate depression was observed in Group 2, in which both white blood cell count (WBC) and packed cell volume (PCV) percentage showed significant decreases. **Conclusion and Clinical Relevance**: The findings of this study suggest that epidural administration of detomidine-lidocaine HCl results in more effective and longer-lasting perineal analgesia compared to xylazine-lidocaine HCl.

## Introduction

The use of a local anesthetic solution for caudal epidural anesthesia in horses was initially documented by Pape and Pitzschk [1]. The technique is a popular method for offering analgesia in the perineal, sacral, lumbar, and caudal regions of the thoracic area due to its ease, affordability, and lack of advanced devices [2]. Caudal epidural analgesia is a widely used technique in equine medicine for diagnostic, clinical, and surgical procedures involving the perineal region [3–5]. This method is particularly advantageous for standing surgeries, as it allows for effective pain management without the risks associated with general anesthesia [2, 6, 7].

Local anesthetics, such as lidocaine, are commonly administered in the caudal epidural space to achieve analgesia [7]. Lidocaine, a fast-acting sodium channel blocker, provides rapid onset of analgesia but is limited by its short duration of action, often requiring re-administration during prolonged procedures [8]. To address this limitation, adjunctive agents such as alpha-2 agonists (e.g., xylazine and detomidine) are increasingly used to extend the duration of analgesia while minimizing side effects [9, 10].

Alpha-2 agonists, including xylazine and detomidine, have gained popularity in veterinary medicine due to their sedative, analgesic, and muscle-relaxant properties. These agents work by stimulating alpha-2 adrenergic receptors in the central nervous system, leading to inhibition of nociceptive transmission and reduced sympathetic outflow [5, 6, 11, 12]. Xylazine, a well-established alpha-2 agonist, has been widely used in horses for perineal analgesia. However, its systemic administration is associated with cardiopulmonary side effects, such as bradycardia, hypotension, and respiratory depression, which may limit its use in certain clinical scenarios [5]. In contrast, detomidine, a more selective alpha-2 agonist, is known for its potent sedative and analgesic effects with fewer cardiovascular side effects, making it a preferred choice for many equine procedures [13]. The combination of lidocaine with alpha-2 agonists has been shown to enhance the quality and duration of epidural analgesia in horses. While lidocaine provides a rapid onset of analgesia, alpha-2 agonists prolong the duration of pain relief by modulating nociceptive pathways in the spinal cord [14].

The current study was prepared to assess the comparative analysis of the effectiveness of xylazine-lidocaine HCl and detomidine-lidocaine HCl following caudal epidural injection in domestic horses (Equus ferus caballus). The findings of this study will help researchers and veterinarians decide which pain management techniques are best for horses receiving caudal epidural injections. Improved treatment procedures and results for horse patients can result from an understanding of the distinctions in efficacy between xylazine-lidocaine HCl and detomidine-lidocaine HCl.

## Materials and methods

### Horse’s collection and injections

A total of fourteen adult (seven males and seven females) horses (*Equus ferus caballus*), aged between 4 and 5 years and weighing 250–300 kg, were selected for this study. Prior to the experiment, the horses were confirmed to be in good health based on clinical and hematological physiological examinations. Exclusion criteria included horses with poor health, body condition scores below 3, an impossible-to-access sacrococcygeal space, a previous history of regional nerve blockage or epidural injections in the perineal area, or skin conditions in the area of interest. The owners’ informed consent was properly obtained, and the horses were taken from their homes right away. Horses were housed in their stalls and fed grass hay enriched with concentrate. They were given unlimited access to water for a full day before the trial, during which time their feed was discontinued [10].

The horses were injected in their stalls. The sedation levels in each horse were classified as follows:

- **Alert**: No sedative effect.
- **Mild sedation**: Reduced alertness without other signs.
- **Moderate sedation**: Drowsiness with minor head drop.
- **Deep sedation**: Significant drowsiness and head drop.

The anatomical terms were applied according to **Nomina Anatomica Veterinaria** [15].

### Experimental study design

The horses were randomly divided into two equal groups, each group included seven horses. Group 1 received xylazine (0.17 mg/kg bwt) + lidocaine HCl (0.06 mg/kg bwt), while Group 2 received detomidine (0.03 mg/kg bwt) + lidocaine HCl (0.06 mg/kg bwt) via epidural injection. The skin over the sacrococcygeal area was surgically prepared before the procedure. Epidural injections were administered in the first intercoccygeal space using an 18-gauge, 5 cm hypodermic needle (AMECO, Egypt). Correct placement in the epidural space was confirmed using the hanging drop technique and the absence of resistance during injection. Following drug administration, the onset of analgesia was assessed using a pin-prick test applied to the anus and perineum to evaluate the level of complete analgesia. The absence of a response to pinpricks was considered indicative of complete analgesia. The presence or absence of a response was recorded for each site and subjectively compared between the two groups.

The study focused on the onset time and duration of complete perineal analgesia. The onset of complete analgesia was defined as the time from injection to the loss of sensation. The duration of complete analgesia was evaluated by testing the response to perineal skin stimulation at baseline (0 min, before injection) and at 15, 30, 60, 90, and 120 min post-injection.

Clinical parameters, including heart rate (HR), respiratory rate (RR), rectal temperature (RT), and hemato-biochemical values, were recorded before and at 15, 30, 60, 90, and 120 min post-injection. Blood samples (both heparinized and non-heparinized) were collected via jugular venipuncture to evaluate red blood cell count (RBC), white blood cell count (WBC), packed cell volume (PCV), and hemoglobin (HB) levels. Also, biochemical parameters, including glucose, cholesterol, creatinine, and urea levels, were measured and analyzed.

### Data analytic technique

Data analysis was performed using GraphPad. A T-test was used to compare the mean ± standard deviation (SD) between groups for the onset and duration of complete analgesia. One-way analysis of variance (ANOVA) was used to detect the changes in HR, RR, RT, glucose, cholesterol, creatinine, and urea levels over the 120-minute sampling period that was also evaluated.

## Results

### Sedation and analgesia

**Fig. 1:**
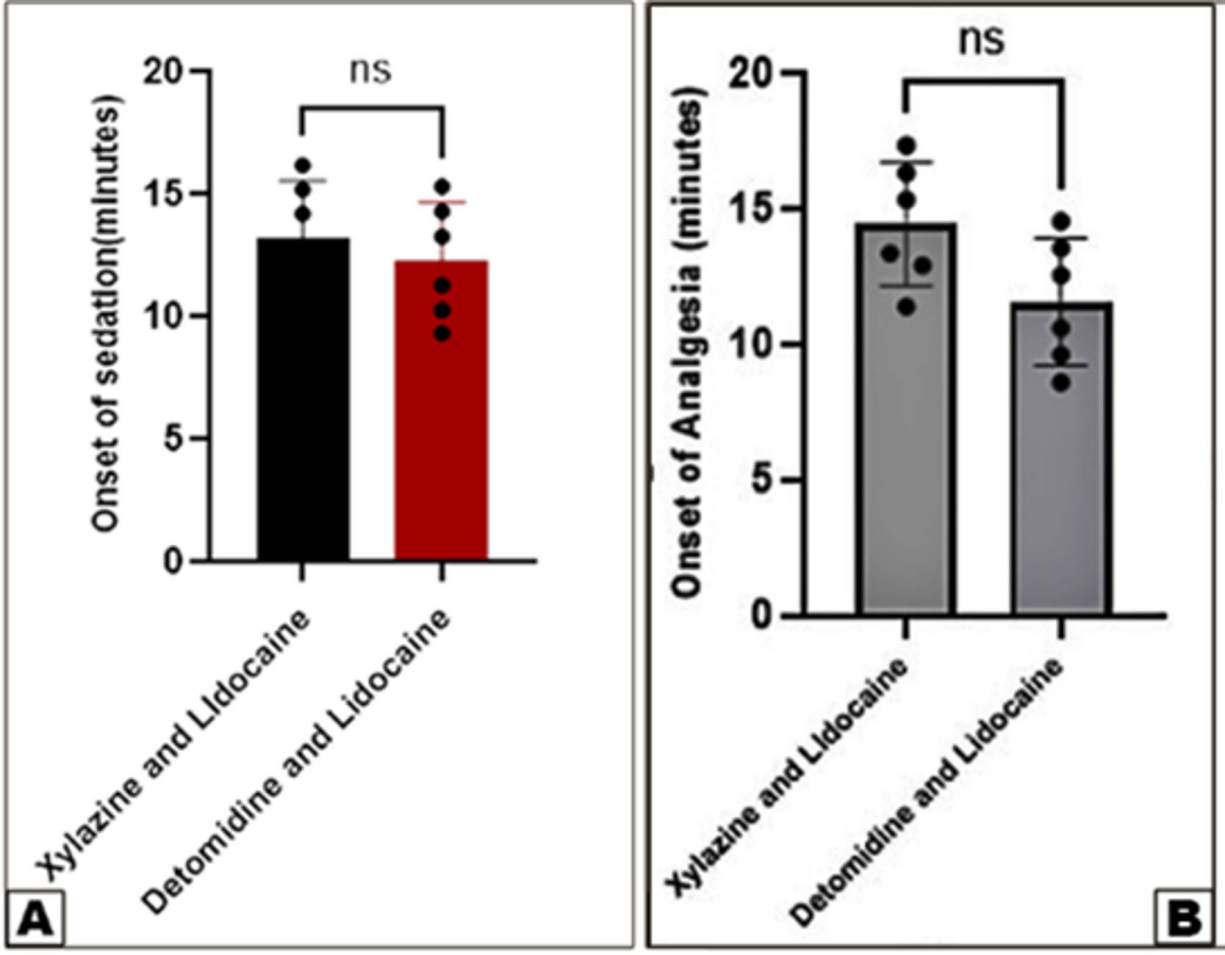
Differences in the onset of sedation (View **A**) and the onset of analgesia (View **B**) between the xylazine-lidocaine and detomidine-lidocaine groups. Data were analyzed using a T-test. Error bars represent the mean ± standard deviation (SD). Means labeled with different letters indicate no significant difference (*p* < 0.05)

**Fig. 2:**
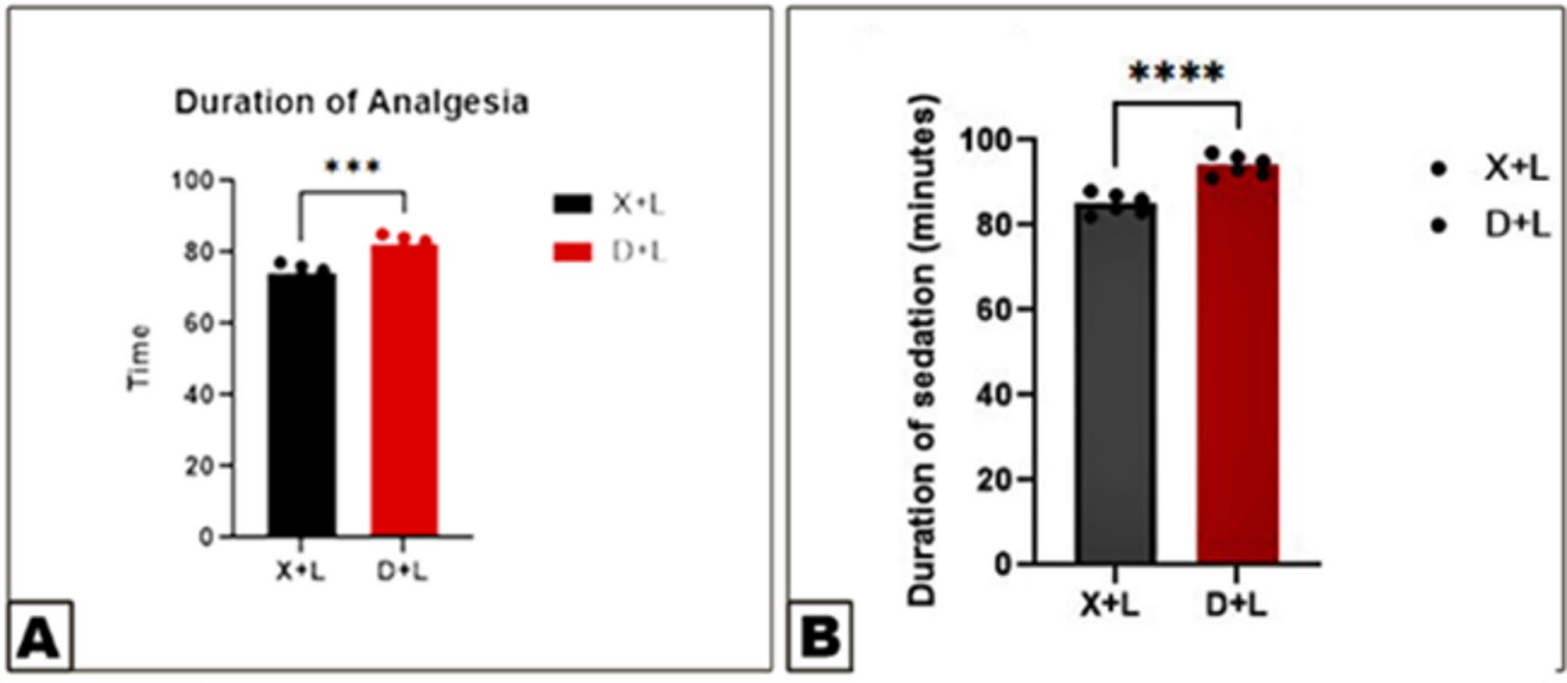
Differences in the duration of sedation (View **A**) and the duration of analgesia (View **B**) between the xylazine-lidocaine and detomidine-lidocaine groups. Data were analyzed using a T-test. Error bars represent the mean ± standard deviation (SD). Means labeled with different letters indicate significant differences (*p* < 0.05)

Detomidine-lidocaine (Group 2) produced a longer duration of sedation compared to xylazine-lidocaine (Group 1), with mean sedation times of 94 ± 0.96 min and 85 ± 0.94 min, respectively. Similarly, the duration of complete perineal analgesia was longer in Group 2 (82 ± 0.93 min) than in Group 1 (74 ± 0.90 min). The onset of analgesia was also slightly faster in Group 2 (11.79 ± 1.15 min) compared to Group 1 (14.46 ± 0.92 min). Both combinations induced complete bilateral perineal analgesia in all horses, as confirmed by the absence of response to pin-prick tests (Figs. 1 and 2).

### Physiological findings

**Fig. 3:**
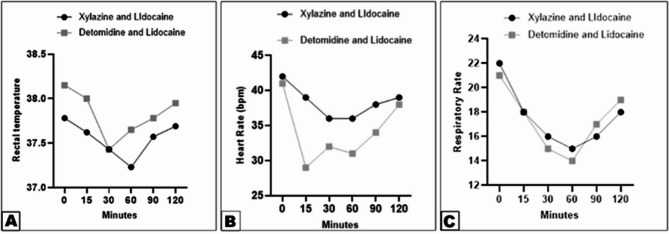
Differences in rectal temperature (View **A**), heart rate (View **B**), and respiratory rate (View **C**) between the xylazine-lidocaine and detomidine-lidocaine groups. Data were analyzed using two-way ANOVA. Error bars represent the mean ± standard deviation (SD). Means labeled with different letters indicate no significant difference (*p* < 0.0001) in rectal temperature (View **A**), while indicating significant differences (*p* < 0.0001) in heart rate (View **B**), and considered significantly different (*p* < 0.0001) in respiratory rate (View **C**)

Rectal temperature (RT) remained stable in both groups, with no significant changes observed (Fig. 3A). Heart rate (HR) decreased significantly in Group 2, with the most pronounced reduction observed at 15–30 min post-injection (Fig. 3B). Respiratory rate (RR) also decreased in both groups, with Group 2 showing a more sustained reduction from 15 to 60 min (Fig. 3C).

### Hematological findings

**Fig. 4:**
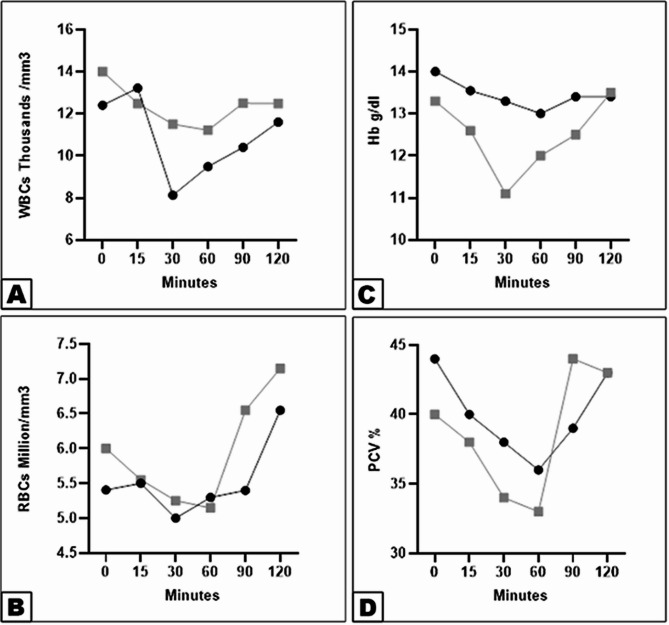
Differences in WBCs rate (View **A**), RBCs (View **B**), Hb (View **C**), and PCV (View **D**) between the xylazine-lidocaine and detomidine-lidocaine groups. Data were analyzed with two-way ANOVA. Error bars represent mean ± SD. Means bearing different letters are considered significantly different (*p* < 0.0001) in WBCs rate (View **A**) and PCV (View D), but are not considered significantly different (*p* < 0.0001) in RBCs (View **B**) and Hb (View **C**)

Hematological analysis revealed significant decreases in white blood cell count (WBC) and packed cell volume (PCV) in both groups. Group 1 showed a more pronounced reduction in WBC at 15–30 min (Fig. 4A), while Group 2 exhibited moderate decreases in PCV from 15 to 60 min (Fig. 4D). Red blood cell count (RBC) and hemoglobin (HB) levels remained within normal physiological ranges in both groups (Fig. 4B-C).

### Biochemical findings

**Fig. 5:**
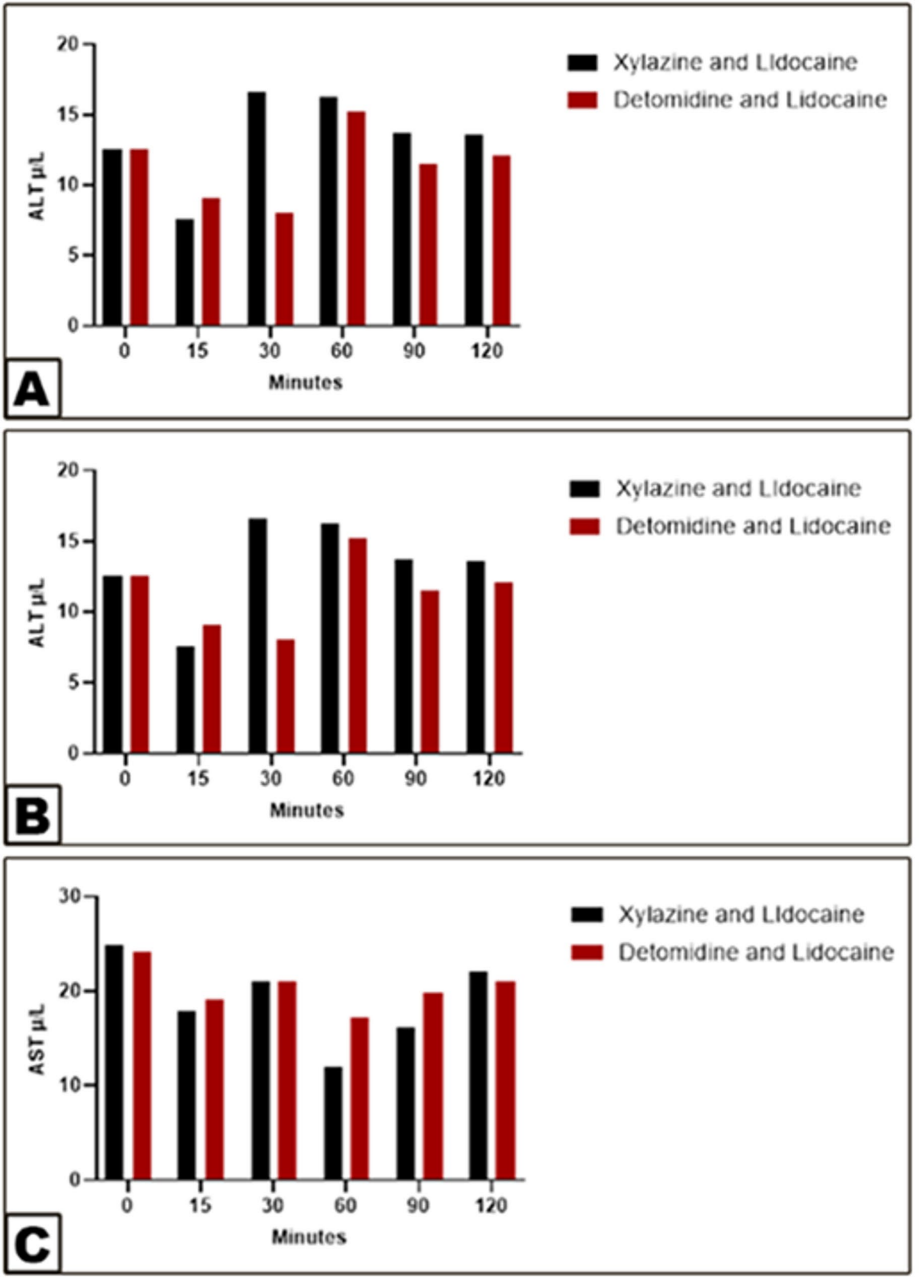
Differences in ALT (View **A**), AST (View **B**), and Total protein (View **C**) between the xylazine-lidocaine and detomidine-lidocaine groups. Data were analyzed with two-way ANOVA. Error bars represent mean ± SD. Means bearing different letters are considered significantly different (*p* < 0.0001) in ALT (View **A**), while they are not considered significantly different (*p* < 0.0001) in AST (View **B**) and Total protein (View **C**)

**Fig. 6:**
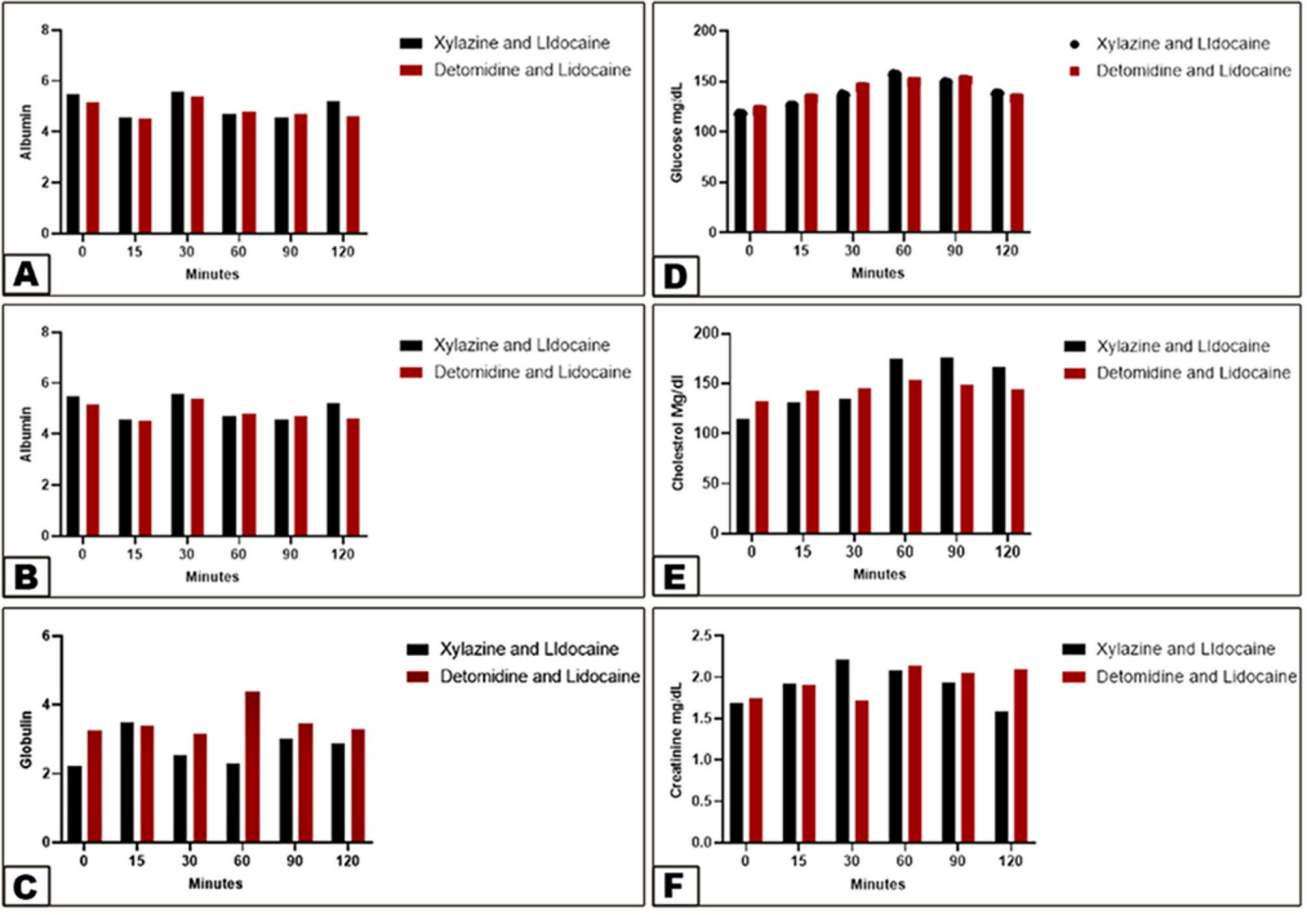
Differences in Albumin (View **A**), Globulin (View **B**), glucose (View **C**), Cholesterol (View **D**), Creatinine (View **E**), and Urea (View **F**) between the xylazine-lidocaine and detomidine-lidocaine groups. Data were analyzed with two-way ANOVA. Error bars represent mean ± SD. Means bearing different letters are not considered significantly different (*p* < 0.0001) in Albumin (View **A**), Globulin (View **B**), glucose (View **C**), and Creatinine (View **E**). While they are considered significantly different (*p* < 0.0001) in Cholesterol (View **D**) and Urea (View **F**)

Significant changes from baseline values in Group 1 were in ALT (decreased by 15 min) and then increased significantly (30 min), while in Group 2 ALT (decreased by 15–30 min), as observed in (Fig. 5A). There were no significant changes in baseline AST, total protein, albumin, globulin, and glucose (Figs. 5B-C and 6A-C). Cholesterol (increased from 60 to 120 min) in group 1, while in group 2, cholesterol (increased from 15 to 60 min) and urea increased from 15 to 90 min, as seen in (Fig. 6F), while no change in creatinine (Fig. 6E).

## Discussion

To perform obstetrical, diagnostic, and surgical procedures in the perineal region of horses, epidural anesthesia is frequently utilized [3, 10, 12]. The current study was prepared to highlight the efficacy of xylazine-lidocaine and detomidine-lidocaine combinations for caudal epidural analgesia in horses, with detomidine-lidocaine demonstrating superior sedative and analgesic properties. The longer duration of sedation (94 ± 0.96 min) and analgesia (82 ± 0.93 min) observed with detomidine-lidocaine, compared to xylazine-lidocaine (85 ± 0.94 min and 74 ± 0.90 min, respectively), can be attributed to detomidine’s high lipophilicity, which facilitates rapid absorption into the cerebrospinal fluid (CSF) and sustained activity at the spinal cord level [16–18].

The current study reveals that the usage of detomidine-lidocaine (Group 2) produced a longer duration of sedation compared to xylazine-lidocaine (Group 1), with mean sedation times of 94 ± 0.96 min and 85 ± 0.94 min, respectively. Similarly, the duration of complete perineal analgesia was longer in Group 2 (82 ± 0.93 min) than in Group 1 (74 ± 0.90 min). In Mediterranean Miniature Donkeys, sedation was longer in the groups that received lidocaine and/or α2-adrenergic agonists than in the group that received lidocaine 45–75 min after drug administration [5, 19].

The current study reveals that the analgesia onset is slightly faster in Group 2 (11.79 ± 1.15 min) than in Group 1, resulting in complete bilateral perineal analgesia in all horses, as confirmed by pin-prick tests. The analgesia onset time following xylazine injection was much longer (32.0 +/- 3.4 min) than the analgesia onset time following lidocaine or the lidocaine/xylazine combination, although the study did not find a significant difference between the two treatments in the horses.

The time to onset of analgesia did not significantly differ between groups of donkeys [19, 20]. There was no discernible difference in the duration of analgesia onset between lidocaine (4.8 ± 1.0 min) and the lidocaine/xylazine combination (5.1 ± 0.9 min). However, the duration of analgesia onset after xylazine was significantly longer (11.7 ± 1.0 min) than either of the other two treatments in cattle [21]. The faster onset of analgesia in the detomidine-lidocaine group (11.79 ± 1.15 min vs. 14.46 ± 0.92 min) further supports its clinical advantage, likely due to its rapid inhibition of substance P release in the dorsal horn of the spinal cord, a key mechanism in nociceptive signaling [22, 23]. These findings align with previous studies reporting the enhanced potency and longer duration of action of detomidine compared to xylazine in equine practice [24]. In comparison to the Mediterranean Miniature Donkeys’ lidocaine group, the duration of analgesia was longer in the groups that received α-2 adrenergic agonists and/or lidocaine [19].

Physiological changes, such as the significant reduction in heart rate (HR) and respiratory rate (RR) in the detomidine-lidocaine group, are consistent with the sympatholytic effects of alpha-2 agonists. By inhibiting norepinephrine release, these agents reduce sympathetic tone, leading to bradycardia and decreased cardiac output [25]. However, these effects were transient and did not result in clinically significant complications, suggesting that detomidine-lidocaine remains a safe option for epidural analgesia in healthy horses. The stability of rectal temperature (RT) in both groups indicates that epidural administration of these agents does not disrupt thermoregulation, unlike systemic administration, which can suppress hypothalamic function [23].

Hematological changes, such as the reduction in white blood cell count (WBC) and packed cell volume (PCV), are likely due to splenic pooling of blood cells and fluid shifts between intravascular and extravascular compartments [26]. These changes are consistent with the stress response and hemodynamic adjustments associated with sedation and analgesia in the mare [27]. Biochemical findings, including transient increases in cholesterol and urea levels, reflect metabolic adjustments during sedation, although these changes remained within physiological limits and did not indicate significant hepatic or renal dysfunction in the horse [28].

## Conclusion

The study evaluated the efficacy of xylazine-lidocaine and detomidine-lidocaine combinations for caudal epidural analgesia in horses, with detomidine-lidocaine showing superior sedative and analgesic properties. In conclusion, detomidine-lidocaine provides more effective and longer-lasting analgesia compared to xylazine-lidocaine, making it a preferable choice for caudal epidural analgesia in horses. These findings offer valuable insights for veterinarians seeking to optimize pain management strategies in equine practice.

## Data Availability

The datasets used and/or analyzed during the current study are available from the corresponding author on reasonable request. Research data are not shared.
